# Development of a Dynamic Multi-Parameter Prediction Model for the Maturation Process of ‘*Ugni Blanc*’ Grapes Using Visible and Near-Infrared Spectroscopy

**DOI:** 10.3390/foods15030475

**Published:** 2026-01-30

**Authors:** Chenxue Su, Jia Che, Zehao Wu, Kai Li, Xiangyu Sun, Yulin Fang, Wenzheng Liu

**Affiliations:** 1 College of Enology, Northwest A&F University, Yangling 712100, China; 2 Xinjiang Research Institute of Agriculture in Arid Areas, Urumqi 830091, China

**Keywords:** Vis/NIR spectroscopy, Ugni Blanc grapes, partial least squares, support vector machine regression, convolutional neural network

## Abstract

In this study, the non-destructive determination of pH, total soluble solids (TSS), total acidity (TA), reducing sugars (RS), seed total phenolic content (TPCD), and skin total phenolic content (TPCN) in Ugni Blanc grapes was performed using visible/near-infrared (Vis/NIR) spectroscopy coupled with chemometric quantitative analysis. Diffuse reflectance spectra in the 400–1507 nm range were measured using a handheld Vis–NIR spectrometer, after which the dataset was partitioned using the SPXY algorithm, accounting for joint X-Y distances. Six spectral preprocessing methods and three modeling algorithms, Partial Least Squares (PLS), Support Vector Machine Regression (SVR), and Convolutional Neural Network (CNN), were used to construct quantitative models based on full-wavelength and feature-wavelength data. Feature-based models outperformed full-spectrum models for TA, RS, and TPCN, whereas full-spectrum models performed better for pH, TSS, and TPCD. The optimal models achieved $R_{p}^{2}$ values of 0.940, 0.957, 0.913, 0.889, 0.917, and 0.871 and *RPD* values of 4.074, 4.798, 3.397, 2.998, 2.904, and 2.786, correspondingly. The findings highlight the applicability of Vis/NIR spectroscopy for the accurate and non-destructive prediction of key physicochemical indicators in Ugni Blanc grapes.

## 1. Introduction

Ugni Blanc is a strategically vital white grape variety in the Cognac region, whose ripeness directly influences the acidity balance, flavor precursor content, and overall quality stability of the distilled spirit [1]. Strict control of ripeness at harvest is crucial for brandy quality: under-ripe grapes produce an astringent profile due to acid–sugar imbalance, while overripe grapes lose aromatic precursors and develop off-flavors, compromising freshness and typical style [2,3,4]. Accurate and rapid monitoring of Ugni Blanc ripeness is vital for optimal harvest. Key indicators include total soluble solids content (TSS), total acidity (TA), and total phenolics content (TPC), which collectively influence potential alcohol, flavor development, freshness, and oxidative stability [5,6,7]. Currently, routine analytical methods for these components generally rely on destructive sampling, which is complex, time-consuming, and costly, and requires trained personnel. These limitations restrict their efficient application in harvest decision-making [8,9].

In recent years, advances in chemometrics and spectroscopic instrumentation have provided new approaches for evaluating the inner quality of fruits without physical damage. Studies have shown that visible–near-infrared (Vis–NIR) spectra in the 350–1800 nm range can effectively predict the TSS of pears [10,11,12]. Fernández-Novales et al. successfully analyzed dynamic changes in amino acids during grape ripening using Vis-NIR spectroscopy [13]. Xiao et al. achieved a classification accuracy of over 77% for grapes based on TSS and TPC using spectra in the 400–1100 nm range [14]. In addition, Yu et al. developed a near-infrared probe that enabled non-destructive prediction of grape TSS [15]. The near-infrared models developed by Basile et al. also demonstrated strong predictive capability for grape quality parameters [16]. Collectively, research confirms that Vis–NIR spectroscopy, applied as a fast and non-invasive technique, has great potential for assessing fruit ripeness and key quality attributes [17,18,19,20].

Despite the broad adoption of Vis–NIR spectroscopy as a non-destructive tool for evaluating fruit ripeness and quality attributes, its combination with deep learning approaches remains insufficiently explored, particularly for the simultaneous prediction of multiple grape quality parameters, including pH, TSS, TA, reducing sugars (RS), and total phenolic compounds present in grape skins and seeds (TPCN and TPCD) [21]. Spectral modeling has often relied on classical machine learning methods, for instance, partial least squares regression (PLS) and support vector regression (SVR), to suppress noise interference and improve prediction accuracy. In recent years, deep learning approaches, particularly convolutional neural networks (CNNs), have advanced rapidly in the field of artificial intelligence, demonstrating excellent feature learning and pattern recognition capabilities and significant potential for extracting and modeling complex spectral information [22]. However, the utilization of neural network techniques for spectral analysis remains relatively limited. Therefore, integrating Vis–NIR spectroscopy with neural network algorithms to systematically enable fast, non-invasive estimation of multiple grape attribute parameters holds significant theoretical value and practical potential [23,24].

This study aimed to evaluate the feasibility of using visible–near infrared (Vis–NIR) spectroscopy combined with multivariate modeling and deep learning methods for rapid, non-destructive assessment of quality parameters in *Ugni Blanc* grapes during ripening. The specific objectives included: (1) collecting Vis–NIR spectra and reference quality data of grapes from véraison to the ripening stage, and developing PLS, SVR, and CNN models; (2) investigating spectral preprocessing methods and characteristic wavelength selection using the successive projections algorithm (SPA) to reduce data dimensionality while maintaining prediction accuracy; and (3) comparing the predictive performance of different models to determine the optimal model for each quality parameter.

## 2. Materials and Methods

### 2.1. Grape Samples

From July to the end of August 2024, *Ugni Blanc* grapes were collected by experienced grape growers in Laizhou, Shandong, China (37°12′3″ N, 120°1′23.9″ E). To account for adequate variability, a stratified sampling technique was employed [25]. To establish fixed sampling points, three rows within the vineyard were randomly selected. For each cluster, grapes were systematically sampled from the top, middle, and bottom sections, and wine grape clusters were partitioned into corresponding strata [26]. Two to three berries were collected from each designated position, resulting in a total of 6–9 berries per grape bunch. Based on grape development characteristics and cultivation experience, samples were collected every 3–5 days, with 500 berries harvested between 8:00 and 10:00 a.m. at each sampling event. Subsequently, the grapes were hand-divided into 8–15 groups, grouping berries with analogous color and firmness [27].

### 2.2. Spectroscopic Measurements

**Figure 1:**
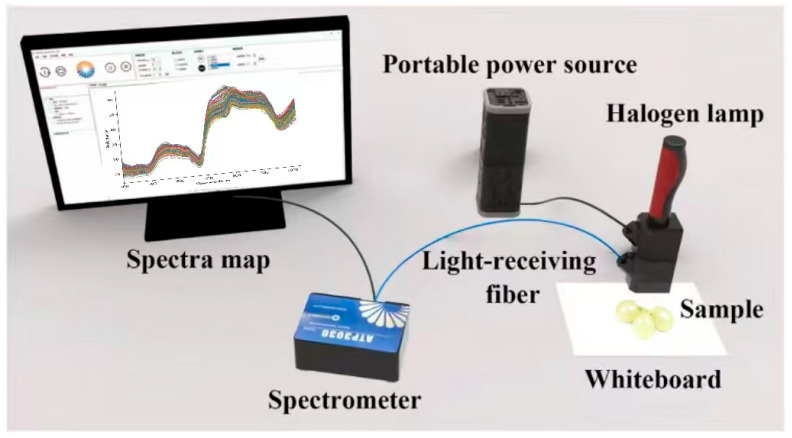
Reflectance spectral acquisition system.

The system utilized a high-resolution miniature spectrometer ATP 3030 (Optosky Photonics Inc., Xiamen, China), equipped with an internal circuit capable of synchronously triggering and controlling the xenon lamp. The spectrometer operates over a wavelength range of 200–1100 nm with a resolution of 0.05–2 nm. All spectral measurements were conducted at an ambient room temperature of approximately 22 ± 2 °C, and the spectrometer was powered on and allowed to warm up for at least 30 min prior to data acquisition to ensure measurement stability and spectral consistency. Other elements of the setup consisted of a 12 V halogen lamp light source (HL 2000), a dark chamber, optical fibers, and a computer equipped with Optosky Spectra V3.1.25 software. A standard whiteboard (Guangzhou Jingyi Optoelectronics Technology Co., Ltd., Xiamen, China) was utilized as the white reference, and calibration was carried out before each measurement according to the defined standard. Each sample underwent ten repeated measurements from its sun-facing and shaded sides [28], and the Euclidean distance was calculated. The spectrum showing the least deviation from other measurements was designated as the representative spectrum for each sample. Grapes were subsequently placed on a standard whiteboard for spectral acquisition. Figure 1 shows a schematic diagram of the spectral acquisition.

### 2.3. Chemical Analysis

#### 2.3.1. Sample Preparation

After completion of spectral measurements, the samples were kept at −40 °C to allow for subsequent reference analyses. The grape skins, pulp, and seeds were then manually separated. The pulp was processed for juice extraction from grapes for the measurable determination of TSS and TA. For phenolic compound analysis, the skins and seeds were first ground and freeze-dried. Phenolics were repeatedly extracted three times with a hydrochloric methanol solution (0.1% *v*/*v* HCl: 60% *v*/*v* methanol), after which the supernatants were merged and stored at −40 °C for further determination [29].

#### 2.3.2. TSS and the Reducing Sugar Content

The TSS of grape juice from each sample was measured using a digital handheld refractometer (PAL-1; Atago Co. Ltd., Tokyo, Japan). RS content was determined using the Fehling reagent hot titration method. Before measurement, the grape juice was prepared by dilution to reach a sugar content within the range of 2–4 g/L [30].

#### 2.3.3. pH and the Total Acid Content

Measurement of the pH in grape samples was conducted using a pH meter (Shanghai, China, Leici, PHS-3E). To determine the TA of each sample, 2 mL of grape juice was analyzed by titration using a standard NaOH solution, with a color change indicator marking the endpoint, and the volume of NaOH consumed was recorded. A blank test was conducted simultaneously for reference [30].

#### 2.3.4. Total Phenolic Content

The total phenolic content in grape skins (TPCN) and seeds (TPCD) was quantified using a slightly modified Folin–Ciocalteu procedure [30,31]. Briefly, 0.1 mL of grape extract was mixed with 0.5 mL of Folin–Ciocalteu reagent and allowed to react for 5 min at room temperature. Subsequently, 1.5 mL of 20% (*w*/*v*) sodium carbonate solution was added, after which the mixture was diluted with distilled water to a final volume of 10 mL. The results were expressed as milligrams of gallic acid equivalents per gram (mg GAE/g).

### 2.4. NIR Spectra Acquisition

#### 2.4.1. Spectral Preprocessing

During the process of spectral acquisition, different types of noise introduced by environmental conditions may compromise both model accuracy and its predictive reliability [32]. Therefore, spectral preprocessing is essential before model development. In this study, six preprocessing methods were applied: Detrending (DT), First Derivative (FD), Multiplicative Scatter Correction (MSC), Savitzky–Golay Smoothing (SG), Standard Normal Variate (SNV), and Standardization or Standard Scaling (SS).

#### 2.4.2. Dataset Partitioning

To ensure that the modeling dataset adequately represented both spectral characteristics and chemical properties of the samples, the SPXY (Sample set Partitioning based on joint X-Y distances) algorithm was implemented to allocate the dataset into training and test groups [33]. The SPXY method considers not only the differences in the spectral space (*X*) but also the distribution of the response variables (*Y*), effectively avoiding uneven sample distribution caused by traditional random partitioning and thereby enhancing model robustness and generalization. In this study, a total of 192 *Ugni Blanc* samples were partitioned into training and test sets at a ratio of 7:3.

#### 2.4.3. Feature Wavelength Selection

Vis–NIR spectral data usually consist of hundreds to thousands of wavelengths, with a large number exhibiting limited correlation with the predicted reference values [34]. Therefore, feature selection of wavelengths is essential for improving the predictive ability of calibration models. In this study, selection of wavelengths was conducted using the Successive Projections Algorithm (SPA).

The SPA method is a sequential projection-based approach for variable selection. It aims to determine the wavelengths that most effectively capture spectral information while minimizing multicollinearity. The algorithm iteratively selects a subset of variables that can maximally capture the spectral information, which are then used for model development [22,35].

#### 2.4.4. Modeling Methods

Three different predictive models were employed in this study: Partial Least Squares Regression (PLS), Support Vector Regression (SVR), and one-dimensional Convolutional Neural Networks (CNNs). The spectral dataset was utilized as input, whereas physicochemical measurements were designated as the target variables.

PLS develops a mathematical relationship connecting spectral variables to the target chemical indicators. By extracting latent variables that maximize the shared covariance between the independent matrix X and the response variable y, PLS compresses high-dimensional spectral data into a limited set of latent variables, controlling model complexity while retaining the main information relevant to the response [36,37]. To determine the optimal latent variable dimensionality, this study employed the integration of a combined fivefold cross-validation with grid-based hyperparameter optimization, in which *n*_components ranged from 1 to the smaller of the number of training samples or spectral variables, allowing automatic selection of the most suitable *n*_components. After fitting the model using the training dataset, it was applied to the test set for prediction, enabling quantitative modeling from spectral data to chemical indicators while effectively addressing the issue of high dimensionality and multicollinearity of the spectral variables.

SVR was used to establish quantitative predictive models between spectral data and target chemical indicators [38]. By employing a kernel function, SVR projects input features into an elevated-dimensional feature representation, where a linear regression-based model is constructed to capture the nonlinear association linking spectral data and response outputs. During model training, key hyperparameters, namely the regularization coefficient *C*, kernel type, and the kernel parameter ϒ, were automatically optimized using randomized search (RandomizedSearchCV) combined with five-fold cross-validation to achieve the best fit. The search was performed over a predefined parameter grid: C = [0.1, 1, 10, 100], ϒ = [‘scale’, ‘auto’, 0.01, 0.1, 1], and kernel types = [‘linear’, ‘rbf’, ‘sigmoid’], with 10 random iterations. Once trained, the model was applied to the test set for prediction, enabling quantitative modeling from spectra to chemical indicators while effectively handling high-dimensional and nonlinear spectral data.

**Figure 2:**
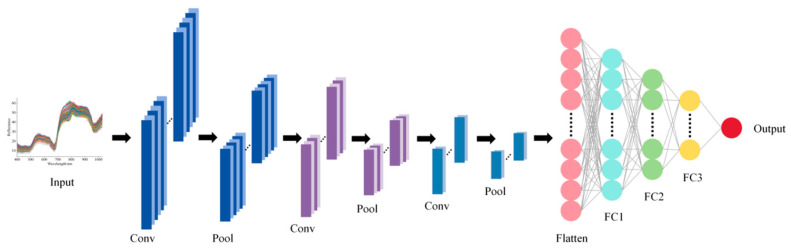
Architecture of CNN model.

A CNN was used to construct regression models for spectral data. The network consists of three convolutional layers, which extract local features from the spectral sequence through convolutional filtering, batch normalization layers, ReLU-based nonlinear activation, and pooling mechanisms. The first convolutional layer has 16 output channels, kernel size 5, and stride 2, followed by max pooling (window 2, stride 1); the second convolutional layer has 32 output channels, kernel size 3, and stride 2, followed by max pooling (window 2, stride 1); the third convolutional layer has 64 output channels, kernel size 1, and stride 1, followed by adaptive max pooling with output length 1. Detailed parameters are listed in Table S1. Figure 2 illustrates the CNN architecture described above. The extracted features are then aggregated into a fixed-length feature vector via adaptive pooling [39]. The convolutional features are subsequently fed into a multilayer fully connected network to map and output continuous response variables: the first fully connected layer has 64 neurons with dropout 0.2, the second has 32 neurons with dropout 0.2, and the output layer has 1 neuron. During training, mean squared error (MSE) served for loss calculation, and network parameters were updated using the Adam optimizer. This network can effectively capture both local and global patterns in spectral data, enabling nonlinear quantitative prediction of high-dimensional spectra.

#### 2.4.5. Evaluation of Model Performance

The performance of the model was comprehensively assessed with several metrics, including the coefficient of determination (*R*^2^), root mean square error (*RMSE*), and residual predictive deviation (*RPD*) [40]. Among them, R^2^ is used to assess how well the predicted values correspond to the measured values [8], calculated as follows:

(1) $$R^{2}=1-\frac{\sum_{i=1}^{n}{\left(y_{i}\;-\;\hat{y_{i}}\right)}^{2}}{\sum_{i=1}^{n}{\left(y_{i}\;-\;\bar{y}\right)}^{2}}$$

where $y_{i}$ is the *i*-th measured value, $\hat{y_{i}}$ is the *i*-th predicted value, $\bar{y}$ is the mean of the measured values, and n is the number of samples. An *R^2^* value closer to 1 indicates stronger predictive ability. *RMSE* reflects the average magnitude of prediction errors and is calculated as follows:

(2) $$\mathrm{RMSE}=\sqrt{\frac{1}{n}\sum_{i=1}^{n}{\left(y_{i}\;-\hat{y_{i}}\right)}^{2}}$$

A smaller *RMSE* value indicates lower prediction error. The *RPD* serves to evaluate the consistency of the model’s predictions and is defined as the quotient of the standard deviation of the response variable to the residuals of the predicted values, calculated as follows:

(3) $$\mathrm{RPD}=\frac{\mathrm{SD}(y)}{\mathrm{RMSE}}=\frac{\sqrt{\frac{1}{n-1}\sum_{i=1}^{n}{\left(y_{i}\;-\;\bar{y}\right)}^{2}}}{\mathrm{RMSE}}$$

In general, RPD values above 2 are indicative of robust predictive performance, while those between 1.4 and 2.0 are acceptable for initial predictions. By comprehensively analyzing these metrics, the performance differences and applicability of the three modeling approaches, PLS, SVR, and CNN, in spectral prediction tasks can be fully compared.

### 2.5. Software and Data Analysis Tools

This study employs different software tools for data processing and visualization, leveraging their respective strengths: data organization and basic statistical analysis utilize Excel software (version 2019; Microsoft Corporation, Washington, DC, USA); complex data analysis, algorithm implementation, and statistical chart generation were performed in the Python environment (Anaconda distribution, Python 3.12), primarily utilizing scientific computing libraries such as Pandas, NumPy, Matplotlib, and Seaborn; and final publication-grade images were refined and generated using Origin 2021 software (OriginLab, Northampton, MA, USA).

## 3. Results and Discussion

### 3.1. Spectral Characteristics of Grapes from Veraison to Maturity

**Figure 3:**
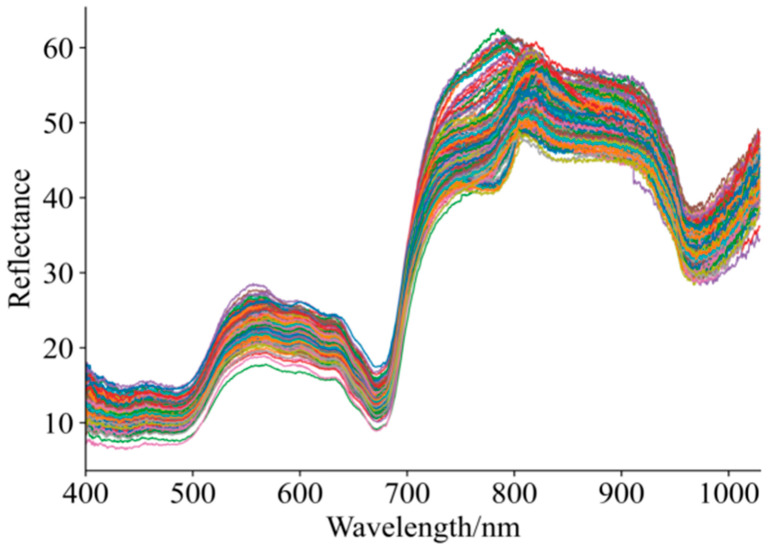
Spectral curves of grapes in ripening period.

Figure 3 shows the diffuse reflectance profiles for 192 grape samples with varying maturity and quality. As shown in Figure 3, although there is overlap and crossover in the unprocessed Vis–NIR spectral profiles of the grape samples, the overall spectral trends are generally consistent. Within the 400–1000 nm spectral range, noticeable differences exist among the curves, which may be attributed to variations in grape maturity affecting skin color and, consequently, the absorption characteristics of the spectra [5,41,42]. Within the 800–920 nm spectral range, absorption bands were observed, which may be related to the absorption of phenolic compounds, cellulose, and sucrose [41,42]. Overall, the dynamic changes in spectral profiles clearly reflect the physiological processes occurring during fruit maturation, including chlorophyll degradation, synthesis of phenolic compounds, and transformation of structural components.

### 3.2. Construction of Full-Wavelength Prediction Models Under Different Preprocessing Methods

**Figure 4:**
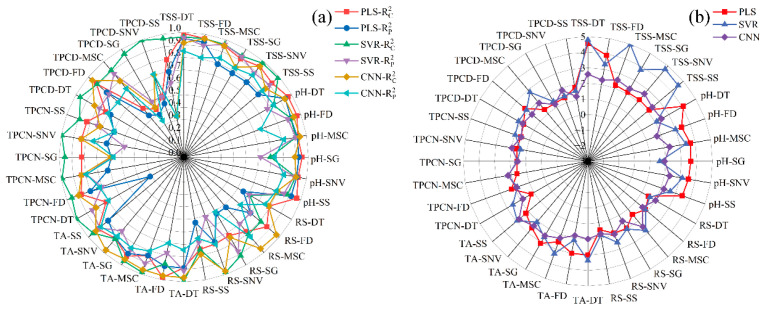
R^2^ and *RPD* values of PLS, SVR and CNN models by using different preprocessing methods. (**a**) Correlation coefficients of the calibration and prediction sets $R_{c}^{2}{,R}_{p}^{2}$; (**b**) Residual Predictive Deviation *RPD*. TSS = total soluble solid content, TA = total acid, RS = reducing sugar, TPCN = total phenolic content of the skin, TPCD = total phenolic content of the seed, PLS = partial least squares, SVR = support vector machine regression, CNN = convolutional neural network.

**Figure 5:**
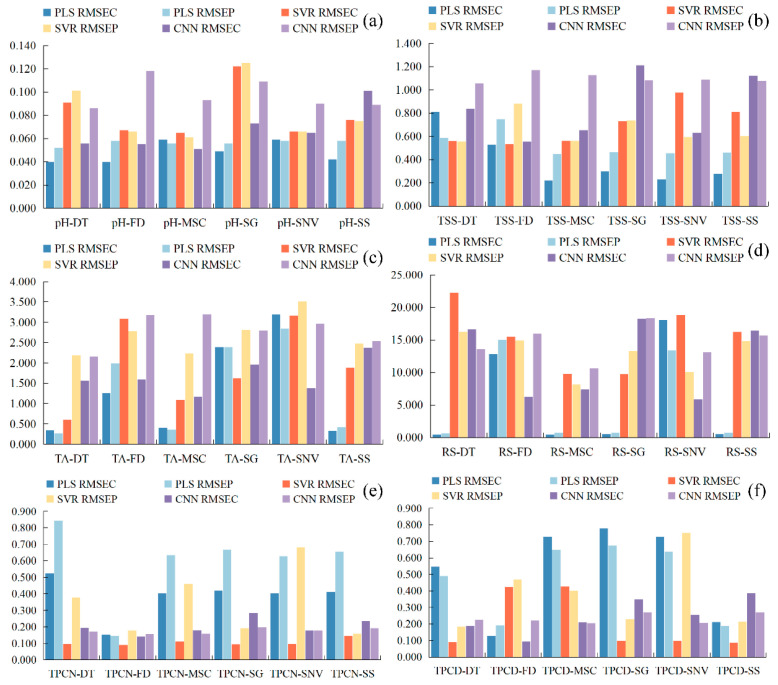
Root mean square error *RMSE* of the correction set. (**a**) pH, (**b**) TSS, (**c**) TA, (**d**) RS, (**e**) TPCN, (**f**) TPCD. TSS = total soluble solid content, TA = total acid, RS = reducing sugar, TPCN = total phenolic content of the skin, TPCD = total phenolic content of the seed, *RMSE* = Root Mean Square Error, PLS = partial least squares, SVR = support vector machine regression, PLS = partial least squares, SVR = support vector machine regression, CNN = convolutional neural network.

Previous studies have shown that visible–near infrared spectroscopy can be employed to predict grape quality attributes, and traditional machine learning approaches have been widely applied to model gluconic acid and other indices in red grape cultivars [25]. Conventional physicochemical analyses, such as measurements of sugars, acids, and phenolic compounds, typically require extensive laboratory procedures, long processing times, and substantial reagent consumption [43]. Therefore, developing a simple and rapid method for determining the physicochemical properties of *Ugni Blanc* grapes is of particular importance. In this study, PLS, SVR, and CNN algorithms were used to construct prediction models for pH, TSS, TA, RS, TPCN, and TPCD based on spectral data subjected to various preprocessing techniques. The model performance metrics (*R*^2^ and *RPD*) are presented in Figure 4, and the *RMSE* values are shown in Figure 5. The details of the sample set division, including the number of samples in calibration and validation sets for each indicator, are provided in Table S2.

As shown in Figure 4 and Figure 5a–c, for the models established for pH, TSS, and TA, all three modeling approaches under different preprocessing methods achieved calibration and validation *R*^2^ values greater than 0.7. Notably, for pH, the *R*^2^ values of both calibration and validation sets exceeded 0.9 across all modeling methods. In addition, the corresponding *RPD* values were higher than 2, in conjunction with low *RMSEC* and *RMSEP* values, indicating strong model reliability and predictive performance [30].

Figure 5d shows that, for RS, the *RMSEC* and *RMSEP* values of the PLS models based on DT, MSC, SG, and SNV preprocessing are consistently lower and similar to each other, indicating that these models capture the general patterns effectively while maintaining strong predictive ability for new samples. Among them, the PLS-SNV model exhibits *R*^2^ > 0.7 for both calibration and test sets, with an *RPD* greater than 1.5, demonstrating good goodness-of-fit and reliable predictive capability. This model is therefore suitable for rapid detection and preliminary quantitative analysis of RS. Other RS models may provide rough quantitative predictions but still show risks of underfitting, making them insufficient for high-precision prediction [44].

For the TPCN, models developed using FD-PLS, DT-SVR, and MSC-CNN demonstrated good performance, achieving *R*^2^ > 0.75. Among the models, FD-PLS achieved the best performance, yielding *R^2^* values of 0.876 and 0.798 for the calibration and test sets, with *RMSEC* and *RMSEP* of 0.153 mg/g and 0.146 mg/g, respectively, and an *RPD* of 2.223, indicating strong predictive capability. These results indicate that the PLS model combined with FD preprocessing not only provides excellent fitting for the training data but also exhibits robust predictive performance for unknown samples [45]. The calibration and prediction sets showed similar performance, with *RMSEP* slightly lower than *RMSEC* [30], indicating that the model did not exhibit overfitting and possesses good generalization ability. An *RPD* exceeding 2.0 further confirms the model’s outstanding quantitative predictive capability, demonstrating its suitability for accurate determination of total phenolics in peel in practical applications [20].

The performance of PLS, SVR, and CNN models in predicting TPCD content varied significantly under different preprocessing methods. PLS was sensitive to preprocessing, with the first derivative (FD) markedly improving model performance, while MSC, SNV, and SG provided limited enhancement. SVR exhibited strong fitting ability but was prone to overfitting under SNV and SG preprocessing; MSC, however, could improve its generalization. CNN demonstrated stable performance across all preprocessing conditions, achieving the best prediction results under FD preprocessing (*RPD* = 4.868), highlighting its superior capability in extracting nonlinear features compared to conventional methods. Overall, FD was identified as the most effective preprocessing approach, and the combination of CNN with FD yielded the optimal balance of predictive accuracy and stability.

Significant differences in prediction performance were observed across multiple quality parameters, including seed total phenolics, peel total phenolics, pH, reducing sugars, total acidity, and SSC, when different preprocessing methods were combined with PLS, SVR, and CNN modeling algorithms. Regarding R^2^, several preprocessing combinations, such as SSC-DT and SSC-SNV, achieved relatively high calibration and prediction coefficients of determination, approaching 0.9. The CNN models showed a clear advantage in capturing complex nonlinear features. Most *RPD* values were relatively high, approaching 5, which indicates strong predictive reliability. In addition, both *RMSEC* and *RMSEP* remained within low ranges, demonstrating small errors in the calibration and prediction sets and reflecting good model stability [46]. Overall, the CNN models displayed superior performance across multiple parameters and proved to be highly suitable for spectroscopic data modeling.

### 3.3. Feature Wavelength Extraction

Owing to the large number of collected reflectance wavelengths (1507) and the strong interdependencies between them, a feature-wavelength selection approach was implemented to enhance the accuracy and computational efficiency of the detection models. Accordingly, SPA was employed to identify key wavelengths. SPA was applied to the optimally preprocessed spectral data to extract informative wavelengths to predict grape pH, TSS, TA, RS, TPCN, and TPCD.

**Figure 6:**
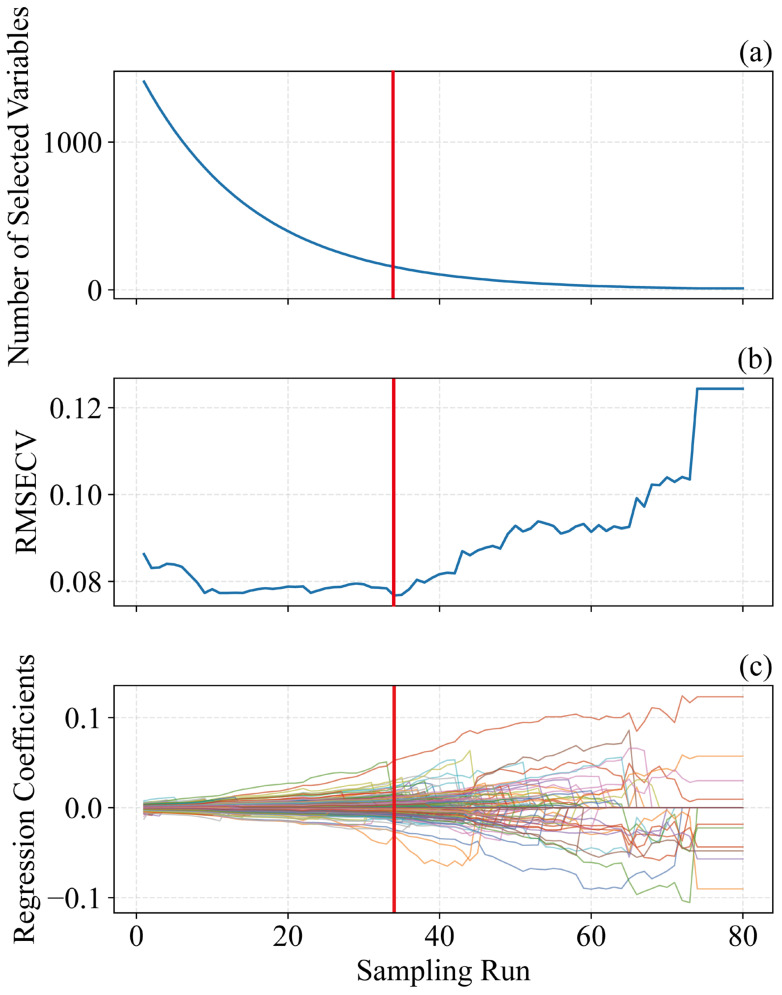
Characteristic wavelength selection charts of pH content in *Ugni Blanc* grape extraction based on SPA algorithm. (**a**) Number of sampled variables, (**b**) 10-fold *RMSEC*V values, (**c**) regression coefficient path of each variable with an increase in the number of sampling runs. SPA = successive projections algorithm, *RMSEC*V = root mean squared error of cross-validation.

Figure 6 illustrates the SPA-based procedure for selecting informative wavelengths, using pH as a case study. In this study, the number of Monte Carlo samplings was set to 80, and a 10-fold cross-validation approach was used to determine the significance of each variable [47]. As shown in Figure 6b, the RMSECV plot reflects the feature selection process by SPA. RMSECV initially decreases and then stabilizes, reaching a minimum at the red line [30]. Figure 6c shows the regression coefficients across iterations, indicating that wavelengths corresponding to the optimal iteration contribute most to the model [20]. With increasing sampling iterations, RMSECV initially declines and then rises: the initial decrease indicates the removal of irrelevant information, while the subsequent increase signals the elimination of useful information [22,35]. Therefore, the sampling run associated with the lowest RMSECV (80 runs) was chosen as the optimal outcome. Based on Figure 6b,c, the optimal iteration occurs at 34 runs, where the regression coefficients of the selected variables align with the vertical line in Figure 6c.

**Figure 7:**
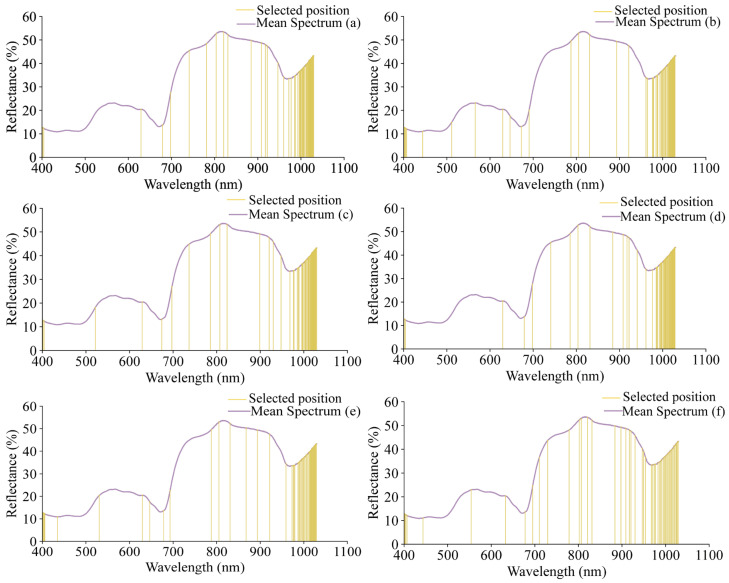
The positions of the characteristic wavelengths of different indicators selected by SPA. (**a**) pH, (**b**) TSS, (**c**) TA, (**d**) RS, (**e**) TPCN, (**f**) TPCD. TSS = total soluble solid content, TA = total acid, RS = reducing sugar, TPCN = total phenolic content of the skin, TPCD = total phenolic content of the seed.

The selected wavelengths are shown in Figure 7. Using SPA, 50 wavelength variables were selected for each of the grape quality indicators (pH, TSS, TA, RS, TPCN, and TPCD), representing 3.3% of the total wavelengths and reducing the data by over 90%. The detailed list of these characteristic wavelengths and their corresponding chemical functional groups is provided in Table S3, offering a comprehensive reference for interpreting the spectral information used in the models.

### 3.4. Construction of Spectral Prediction Models Based on Feature Wavelengths

**Figure 8:**
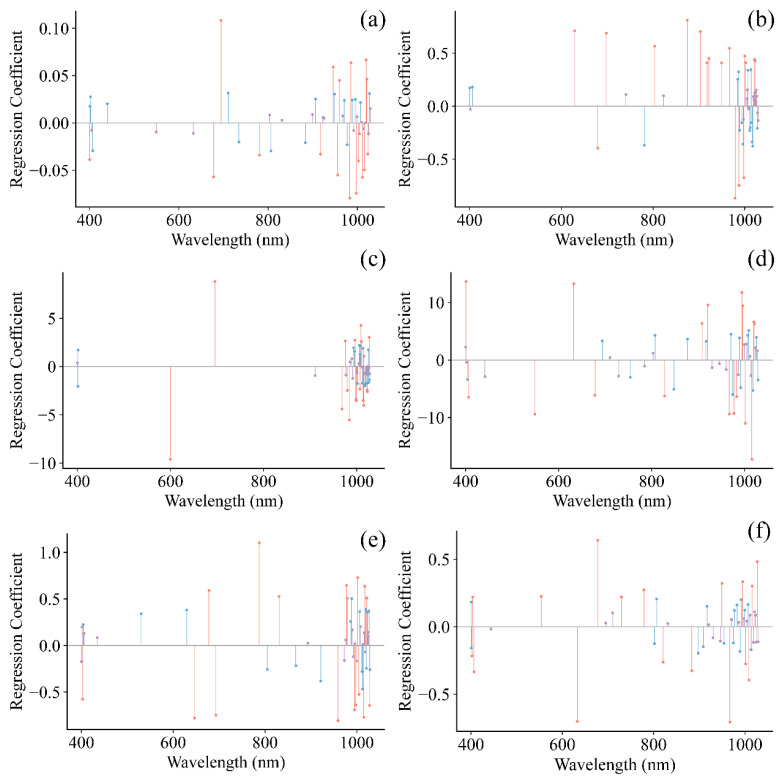
Regression coefficients of PLS model. (**a**) pH, (**b**) TSS, (**c**) TA, (**d**) RS, (**e**) TPCD, (**f**) TPCN. TSS = total soluble solid content, TA = total acid, RS = reducing sugar, TPCD = total phenolic content of the seed, TPCN = total phenolic content of the skin.

The SPA method was used to identify key spectral regions, minimizing the dimensionality of the input data and facilitating the development of more robust and streamlined models [22,35]. Following the selection of informative wavelengths for grape pH, TSS, TA, RS, TPCN, and TPCD, the spectral data were employed to build models using PLS, SVR, and CNN. Figure 8 shows the regression coefficients of the established PLS models, where the red lines indicate wavelengths with high influence on the quality indicators (top 25%), blue lines represent moderately influential wavelengths (middle 50%), and purple lines denote wavelengths with low influence (bottom 25%).

**Figure 9:**
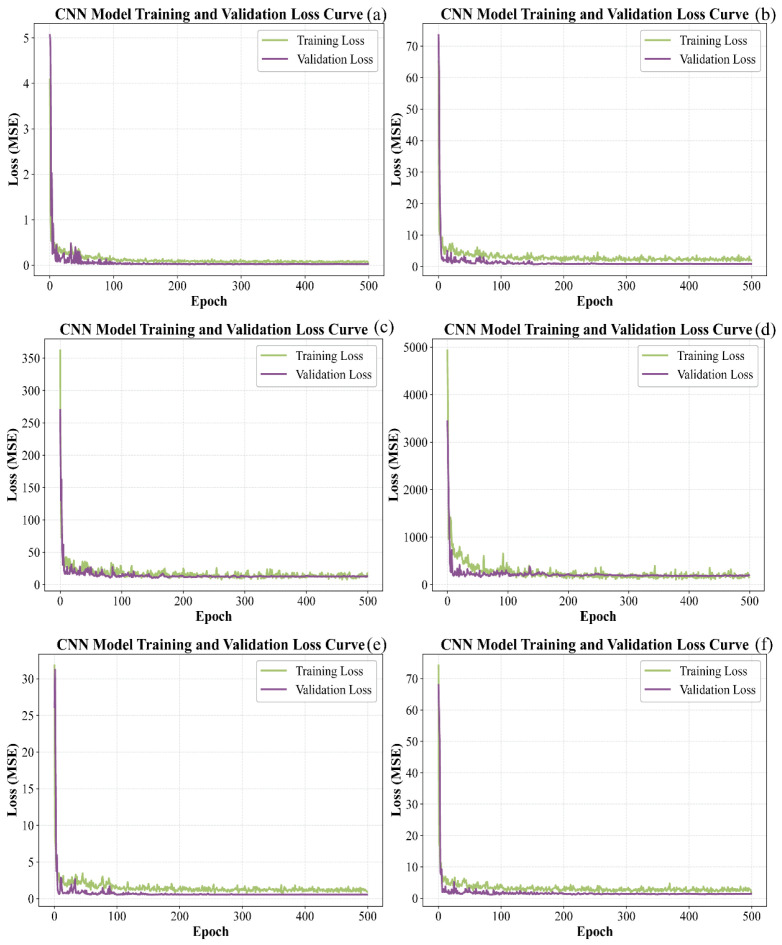
Loss curves of CNN models by calibration set and prediction set. (**a**) pH, (**b**) TSS, (**c**) TA, (**d**) RS, (**e**) TPCN, (**f**) TPCD. TSS = total soluble solid content, TA = total acid, RS = reducing sugar, TPCD = total phenolic content of the seed, TPCN = total phenolic content of the skin, CNN = convolutional neural network.

Figure 9 shows the progression of loss for CNN models applied to the calibration and prediction subsets. It is evident that the loss for all six models decreased rapidly at the initial stage, then declined more slowly and exhibited some fluctuations. After 500 iterations, the loss for the training and test datasets gradually stabilized. For pH, the loss values of both sets approached zero. For TSS, TA, TPCN, and TPCD, the prediction set loss values approached zero while the calibration set loss values remained slightly higher. In contrast, the calibration and prediction set loss values for RS remained relatively high.

**Figure 10:**
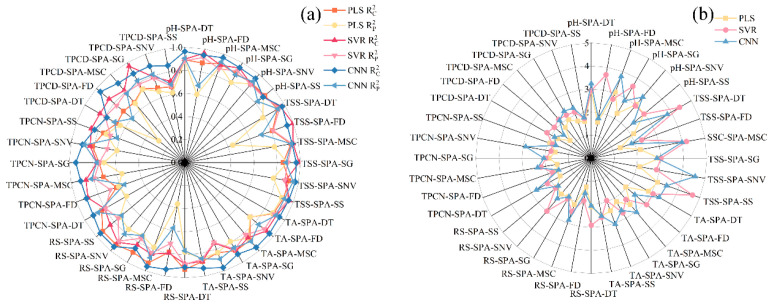
Comparison of model performance (*R*^2^ and *RPD*) using feature wavelengths. (**a**) Correlation coefficients of the calibration and prediction sets $R_{c}^{2},\;R_{p}^{2}$, (**b**) Residual Predictive Deviation *RPD*. TSS = total soluble solid content, TA = total acid, RS = reducing sugar, TPCN = total phenolic content of the skin, TPCD = total phenolic content of the seed, PLS = partial least squares, SVR = support vector machine regression, CNN = convolutional neural network.

**Figure 11:**
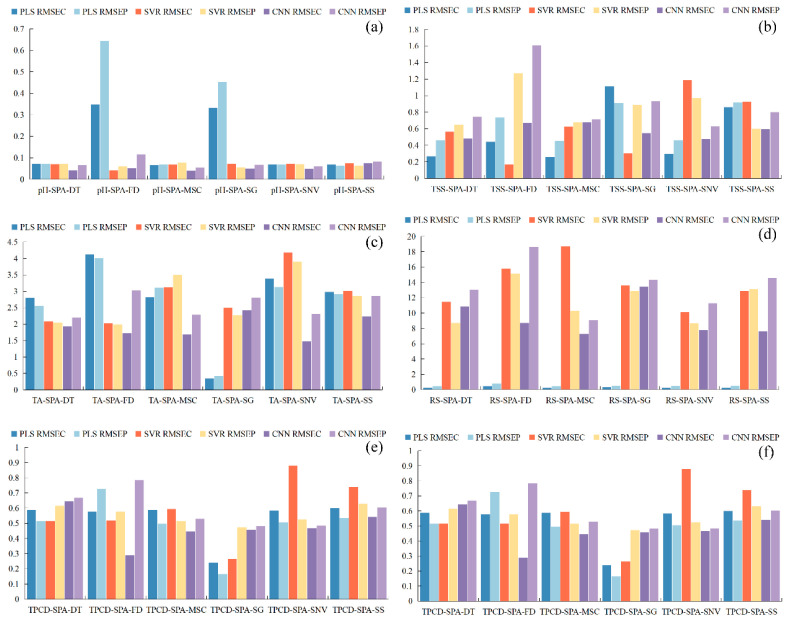
Performance comparison of PLS, SVR, and CNN models based on feature wavelengths using *RMSE*. *RMSE* = Root Mean Square Error, (**a**) pH, (**b**) TSS, (**c**) TA, (**d**) RS, (**e**) TPCN, (**f**) TPCD. TSS = total soluble solid content, TA = total acid, RS = reducing sugar, TPCN = total phenolic content of the skin, TPCD = total phenolic content of the seed, PLS = partial least squares, SVR = support vector machine regression, CNN = convolutional neural network.

The performance metrics of the PLS, SVR, and CNN models developed from the selected feature wavelengths for pH, SSC, TA, RS, TPN, and TPD are shown in Figure 10 and Figure 11. The findings indicate that model performance using the selected feature wavelengths for pH, SSC, and TA was generally superior to that for RS, TPN, and TPD. However, for pH, SSC, and TPD, the prediction accuracy of all three models decreased compared with the full-wavelength models after feature wavelength selection. In contrast, for TA, RS, and TPN, the three models exhibited improved prediction performance after selecting key wavelengths relative to the models based on the full spectrum.

Analysis of the quantitative spectral prediction models for pH, TSS, and TPCD (Figure 10 and Figure 11a,b,f) showed that the overall prediction accuracy decreased after SPA-based feature wavelength selection. For pH, the PLS model yielded $R_{c}^{2}$ values of 0.877–0.910 and $R_{p}^{2}$ values of 0.600–0.893; the SVR model achieved $R_{c}^{2}$ values of 0.890–0.963 and $R_{p}^{2}$ values of 0.864–0.926; and the CNN model showed $R_{c}^{2}$ values of 0.885–0.968 and $R_{p}^{2}$ values of 0.678–0.930. Although predictive performance slightly decreased relative to the full-spectrum models, the CNN model still maintained the highest fitting accuracy and strong robustness, with most RPD values exceeding 3. For TSS, the overall model accuracy also declined after SPA selection. The CNN model remained the best performer, with $R_{c}^{2}$ = 0.972, $R_{p}^{2}$ = 0.952, and an RPD of 4.565. However, its performance was still marginally inferior to that of the full-wavelength model, suggesting that excessive wavelength compression may compromise the completeness of the spectral information. TPCD exhibited a similar trend. Although the CNN model achieved an $R_{c}^{2}$ of up to 0.955, the prediction performance decreased, with $R_{p}^{2}$ values of only 0.593–0.820. Compared with the full-wavelength model, this decline indicates that feature selection reduces redundant wavelengths but may also remove critical spectral information, ultimately limiting prediction accuracy [22].

For TA, RS, and TPCN (Figure 10 and Figure 11c–e), all three models constructed using feature wavelengths showed improved performance compared with the full-wavelength models. In predicting TA, the CNN model outperformed the other models, with *R*^2^ values of 0.970 and 0.913 for the calibration and prediction sets, respectively, an *RPD* of 3.397, and minimal prediction error. These results demonstrate that deep feature extraction combined with wavelength selection provides a clear advantage for total acidity prediction [22]. For RS, the PLS model performed less effectively on the prediction set ($R_{p}^{2}$: 0.364–0.776; RPD: 1.254–2.111) compared with the SVR model ($R_{p}^{2}$: 0.713–0.889; RPD: 1.867–2.998). The CNN model again achieved the best predictive performance ($R_{p}^{2}$: 0.576–0.878; RPD: 2.860). These findings indicate that feature-wavelength selection can effectively extract key information and remove redundant variables, enabling the construction of more efficient and reliable predictive models for RS than full-wavelength approaches [20]. A similar behavior was noted in the prediction of TPCN. The PLS model ($R_{p}^{2}$: 0.553–0.724; RPD: 1.495–1.903) showed relatively weak performance, while the SVR model ($R_{p}^{2}$: 0.682–0.793; RPD: 1.773–2.904) performed better. Once again, the CNN model exhibited superior predictive accuracy and robustness, achieving $R_{p}^{2}$ values ranging from 0.625 to 0.880, *RPD* values between 2.051 and 2.883, and the lowest RMSEP [30].

The results indicate that the key wavelengths selected by SPA provided effective information for all three modeling approaches [22,35]. The CNN models were able to capture complex nonlinear relationships more fully when processing these high-dimensional features, whereas PLS was constrained by its linear nature, and SVR showed slightly lower prediction accuracy than CNN for certain samples. These findings demonstrate that, when applied to feature-selected high-dimensional spectral data, convolutional neural networks offer clear advantages in nonlinear mapping and modeling robustness [30,35].

### 3.5. Determination of the Optimal Models

Based on the results shown in Figure 3 and Figure 9, the prediction models developed for the six quality parameters were comprehensively analyzed. For pH, the full-wavelength PLS model built using DT-preprocessed spectra was identified as the optimal model, with $R_{c}^{2}$, $R_{p}^{2}$ and RPD values of 0.967, 0.940, and 4.074, respectively. For SSC, the SVR model constructed after DT preprocessing exhibited the best performance, achieving $R_{c}^{2}$ = 0.963, $R_{p}^{2}$ = 0.957, and an RPD of 4.798. For TPCN, the SVR model performed best when MSC-preprocessed spectra were used, yielding $R_{c}^{2}$ = 0.907, $R_{p}^{2}$ = 0.871, and an RPD of 2.786. For TA, the best-performing model was the CNN model constructed using DT-preprocessed spectra, followed by SPA-based feature wavelength selection, with $R_{c}^{2}$ = 0.946, $R_{p}^{2}$ = 0.913, and an RPD of 3.397. For RS, the most effective model was identified as the SVR model developed from SNV-preprocessed spectra combined with feature wavelength selection, achieving $R_{c}^{2}$ = 0.903, $R_{p}^{2}$ = 0.889, and an RPD of 2.998. For TPN, the optimal model was again an SVR model, constructed using MSC-preprocessed spectra together with feature wavelength selection, resulting in $R_{c}^{2}$ = 0.917, $R_{p}^{2}$ = 0.907, and an RPD of 2.904.

**Figure 12:**
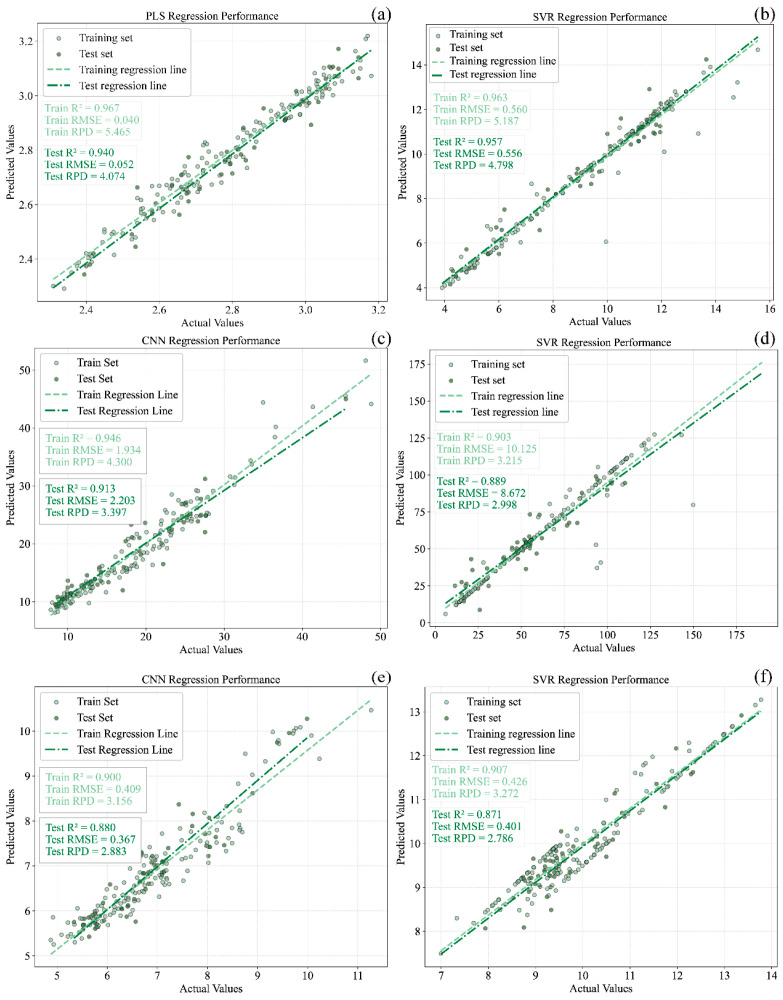
The best models of pH, TSS, TA, RS and total phenols in grape samples. (**a**) pH, (**b**) TSS, (**c**) TA, (**d**) RS, (**e**) TPCN, (**f**) TPCD. TSS = total soluble solid content, TA = total acid, RS = reducing sugar, TPCN = total phenolic content of the skin, TPCD = total phenolic content of the seed, PLS = partial least squares, SVR = support vector machine regression, CNN = convolutional neural network.

Scatter plots illustrating the correspondence between predicted and reference values for the best calibration and prediction models of pH, TSS, TA, RS, TPCN, and TPCD in grape samples are presented in Figure 12. The CNN-based deep learning approach employed in this study is capable of autonomously learning and extracting features through multiple convolutional layers. Previous spectroscopic studies have demonstrated that CNNs often exhibit superior predictive performance compared with traditional machine learning methods [30,48,49]. After key feature wavelengths were selected, the CNN model exhibited marginally superior performance to PLS and SVR in predicting TA in *Ugni Blanc* grapes. This improvement may be attributed to the ability of CNNs to capture complex nonlinear relationships and their inherent robustness to noise arising from illumination or sample variability. The feature wavelength selection further reduced data redundancy and noise, providing a more concise input for the CNN model and thereby improving prediction accuracy.

For the optimal prediction models of the six physicochemical parameters, all $R_{p}^{2}$ values exceeded 0.85, and those for pH, TSS, TA, and TPCN were above 0.90, indicating strong predictive capability. However, the CNN model in this study was deliberately kept shallow, as spectral data are one-dimensional, and overly deep networks tend to cause overfitting, whereas deeper neural architectures typically perform better on larger datasets.

## 4. Conclusions

The raw spectral data were first subjected to six preprocessing methods for this analysis, followed by modeling using PLS, SVR, and CNN. The results showed that for pH, TSS, and TA, the models based on DT-preprocessed spectra performed best, while the prediction accuracy of TPCN and TPCD was significantly improved after MSC preprocessing. For RS, model performance was enhanced following SNV preprocessing. Further, feature wavelengths were extracted from the optimally preprocessed spectra using the SPA algorithm. For each parameter, 50 feature wavelengths were extracted, representing only 3.3% of the full spectral range and reducing the data volume by over 95%. A comprehensive comparison of PLS, SVR, and CNN models constructed with both full-spectrum and selected feature wavelengths revealed that for TA, RS, and TPCN, feature wavelength selection not only substantially reduced computational cost but also improved prediction accuracy. In contrast, model accuracy for pH, TSS, and TPCD showed a slight decline. The optimal models for each parameter were determined as follows: DT-PLS (pH), DT-SVR (TSS), DT-SPA-CNN (TA), SNV-SPA-SVR (RS), MSC-SPA-SVR (TPCN), and MSC-SVR (TPCD). The corresponding prediction set evaluation metrics were: $R_{p}^{2}$ = 0.940, 0.957, 0.913, 0.889, 0.917, and 0.871; RMSEP = 0.052, 0.585; Brix, 2.203 g/L, 8.672 g/L, 2.904 mg/g, and 0.401 mg/g; RPD = 4.074, 4.798, 3.397, 2.998, 2.904, and 2.786, respectively. Despite the promising results, the applicability of these models to other grape varieties remains to be validated. Additionally, the current study only includes samples from a single vineyard and a single season, which may limit the generalizability of the models. Future work should incorporate samples from multiple sources and harvest years to enhance the robustness and reliability of the predictive models.

## 5. Future

For future research, the collection of grape samples could be further expanded to include samples from different sources and years, enhancing model comprehensiveness. Additionally, fine-tuning and training models using multi-year datasets could improve model stability and generalizability. This would allow for deeper CNN architectures, further increasing model robustness and predictive accuracy and promoting the use of deep learning in this field.

## Data Availability

The original contributions presented in this study are included in the article/Supplementary Materials. Further inquiries can be directed to the corresponding authors.

## Supplementary Materials

The following supporting information can be downloaded at: https://www.mdpi.com/article/10.3390/foods15030475/s1, Table S1. Layers and parameters of the one-dimensional convolutional neural network architecture. Table S2. Dataset Division Using SPXY Method. Table S3. Characteristic wavelengths of grape quality indicators and their corresponding chemical functional groups.
