# Supplementary material for: Development of a Dynamic Multi-Parameter Prediction Model for the Maturation Process of ‘Ugni Blanc’ Grapes Using Visible and Near-Infrared Spectroscopy
Source: Foods. 2026 Jan 30;15(3):475. doi: 10.3390/foods15030475 (PMC12897312; doi:10.3390/foods15030475)
Supplement: Supplementary file 1 [file foods-15-00475-s001.zip › foods-4095324-supplementary.pdf]

Table S1. Layers and parameters of the one-dimensional convolutional neural network architecture.

Layers	Parameters	Activation	Additional Processing
Convolution-1D (1)	Kernel number = 16<br>Kernel size = 5<br>Stride = 2	ReLU	Batch Normalization
Max Pooling (1)	Pool size = 2<br>Stride = 1	—	—
Convolution-1D (2)	Kernel number = 32<br>Kernel size = 3<br>Stride = 2	ReLU	Batch Normalization
Max Pooling (2)	Pool size = 2<br>Stride = 1	—	—
Convolution-1D (3)	Kernel number = 64<br>Kernel size = 1<br>Stride = 1	ReLU	Batch Normalization
Adaptive Max Pooling	Output size = 1	—	Global feature aggregation
Flatten	—	—	Feature vectorization
Dense (1)	Neurons = 64	ReLU	Dropout = 0.2
Dense (2)	Neurons = 32	ReLU	Dropout = 0.2
Dense (3) (Output)	Neurons = 1	—	Regression output

Table S2. Dataset Division Using SPXY Method.

Physicochemical parameters	Dataset	Max	Min	Average
pH	training set	3.18	2.31	2.79
	test set	3.15	2.40	2.80
TSS °Brix	training set	15.55	3.90	8.96
	test set	13.65	4.20	9.18
TA g/L	training set	48.80	7.84	19.52
	test set	45.56	8.34	16.12
RS g/L	training set	189.83	6.00	62.38
	test set	111.17	12.67	55.35
TPCN mg/L	training set	3.84	1.62	2.36
	test set	3.26	1.70	2.24
TPCD mg/L	training set	4.55	2.33	3.37
	test set	4.53	2.79	3.30

Table S3. Characteristic wavelengths of grape quality indicators and their corresponding chemical functional groups.

Physicochemical parameters	Wavelength Range (nm)	Functional Groups
pH	400-500	O-H, C-H, Aromatic
	580-700	O-H, C-H
	700-820	O-H, C-H
	900-1030	O-H, C-H
TSS	400-402	Aromatic, C=C
	695	O-H, C-H
	910	O-H, C-H
	968-993	O-H, C-H
	995-1028	O-H, C-H
TA	400-405	Aromatic, C=C
	500-700	O-H, C-H
	700-820	O-H, C-H
	820-1030	O-H, C-H
RS	400-405	Aromatic, C=C
	629-698	O-H, C-H
	740-831	O-H, C-H
	884-921	O-H, C-H
	940-961	O-H, C-H
	976-1028	O-H, C-H
TPCN	400-450	Aromatic, C=C
	633-694	O-H, C-H
	734-823	O-H, C-H
	843-898	O-H, C-H
	913-947	O-H, C-H
	972-1029	O-H, C-H
TPCD	400-402	Aromatic, C=C
	695	O-H, C-H
	838-910	O-H, C-H
	968-993	O-H, C-H
	995-1005	O-H, C-H
	1007-1028	O-H, C-H
